# A Flippase-Mediated GAL80/GAL4 Intersectional Resource for Dissecting Appendage Development in *Drosophila*

**DOI:** 10.1534/g3.115.019810

**Published:** 2015-08-13

**Authors:** Brittany N. Smith, Arash M. Ghazanfari, Rudolf A. Bohm, William P. Welch, Bing Zhang, John P. Masly

**Affiliations:** * Department of Biology, University of Oklahoma, Norman, Oklahoma 73019; † Department of Biological and Health Sciences, Texas A&M University, Kingsville, Texas 78363; ‡ Division of Biological Sciences, University of Missouri, Columbia, Missouri 65211

**Keywords:** *Drosophila*, enhancer-trap Flippase, imaginal discs, genetic manipulation

## Abstract

*Drosophila* imaginal discs provide an ideal model to study processes important for cell signaling and cell specification, tissue differentiation, and cell competition during development. One challenge to understanding genetic control of cellular processes and cell interactions is the difficulty in effectively targeting a defined subset of cells in developing tissues in gene manipulation experiments. A recently developed Flippase-induced intersectional GAL80/GAL4 repression method incorporates several gene manipulation technologies in *Drosophila* to enable such fine-scale dissection in neural tissues. In particular, this approach brings together existing *GAL4* transgenes, newly developed enhancer-trap *flippase* transgenes, and *GAL80* transgenes flanked by Flippase recognition target sites. The combination of these tools enables gene activation/repression in particular subsets of cells within a *GAL4* expression pattern. Here, we expand the utility of a large collection of these enhancer-trap *flippase* transgenic insertion lines by characterizing their expression patterns in third larval instar imaginal discs. We screened 521 different enhancer-trap *flippase* lines and identified 28 that are expressed in imaginal tissues, including two transgenes that show sex-specific expression patterns. Using a line that expresses Flippase in the wing imaginal disc, we demonstrate the utility of this intersectional approach for studying development by knocking down gene expression of a key member of the planar cell polarity pathway. The results of our experiments show that these enhancer-trap *flippase* lines enable fine-scale manipulation in imaginal discs.

Understanding the mechanistic basis of morphogenesis is a major goal of developmental biology. Among the various experimental systems that have been the focus of studying morphogenesis, the imaginal discs of *Drosophila melanogaster* have proven to be particularly valuable in furthering our understanding of several developmental processes that contribute to the formation of adult body parts. Imaginal disc tissues derive from precursor cells that are specified early during embryonic development via invagination of the embryonic ectoderm. These cells proliferate during each of the three larval instar stages to form morphologically distinct tissues, then differentiate during pupation in response to the steroid hormone 20-hydroxyecdysone to give rise to the adult appendages and other parts of the head, thorax, and abdomen (Ursprung and Nöthiger 1972). Studies of imaginal disc biology have made significant contributions to axis specification and patterning (Estella *et al.* 2012; Singh *et al.* 2012), induction and signal transduction (Ramírez-Weber and Kornberg 2000; Swarup and Verheyen 2012), cell fate specification and differentiation (Furman and Bukharina 2012; Treisman 2013), cell growth and proliferation (Wartlick *et al.* 2011; Baena-Lopez *et al.* 2012), cell and tissue polarity (Mlodzik 1999; Müller 2000), and sex determination (Sánchez and Guerrero 2001; Estrada *et al.* 2003). Research using imaginal discs has also proven fruitful for understanding other interesting aspects of development including cell competition (Morata and Martín 2007; Zoranovic *et al.* 2013), coordination of organ growth (Shingleton 2010; Andersen *et al.* 2013), and medically related processes such as regeneration (Belacortu and Paricio 2011; Repiso *et al.* 2011; Worley *et al.* 2012) and tumorogenesis (Pastor-Pareja and Xu 2013; Amoyel *et al.* 2014).

Many of these discoveries have been made possible by advances in transgenic gene manipulation technologies that have provided increasingly fine-scale methods of dissecting morphogenetic processes (Venken and Bellen 2007; del Valle Rodriguez *et al.* 2012). In particular, the GAL4-UAS system has made cell and tissue-specific manipulation of gene expression possible in a variety of tissues and developmental stages (Duffy 2002). In this approach, a transgene containing the open reading frame (ORF) of the *Saccharomyces cerevisiae* transcriptional activator GAL4 is expressed under the control of a tissue-specific regulatory region from either a known fly gene or from an enhancer trap. Expression of the GAL4 protein activates expression of a transgenic target ORF that lies downstream of the GAL4 binding site (*i.e.*, upstream-activating sequence, *UAS*). Thousands of enhancer-GAL4 and UAS transgenic lines exist and the GAL4-UAS method is arguably the most widely used genetic manipulation technique in *Drosophila*.

Although the available GAL4-UAS resources make possible targeted gene expression studies, most enhancer-GAL4 expression patterns are often rather broad in developing tissues, which makes it difficult to perform greater-resolution studies of cell interactions. As developmental studies become increasingly focused on understanding interactions among specific subsets of cells, the need exists to obtain even finer cellular-level resolution to further dissect developmental processes and avoid potential pleiotropic effects that can confound interpretation of experimental results. Several techniques have been developed to target specific cells more precisely for gene manipulation experiments (reviewed in Fore and Zhang 2014). These include approaches such as “split-GAL4” (Luan *et al.* 2006), an intersectional method in which the GAL4 DNA-binding and activation domains are expressed separately under the control of different enhancers to express GAL4 only in the domain of overlap, the construction of a large collection enhancer-trap lines that express *S. cerevisiae*−derived GAL80 to repress the activity of GAL4 within a particular enhancer-GAL4 expression pattern (Suster *et al.* 2004), and the binary Q system, which has been adapted from *Neurospora crassa* to enable gene expression and repression similar to the GAL4-GAL80 system (Potter *et al.* 2010).

Recently, a **F**lippase-induced **in**tersectional **G**AL80/GAL4 **r**epression (FINGR) method was pioneered to map neural circuits in *Drosophila* and brings together several genetic technologies that allow researchers to either activate or repress GAL4 activity in a specific subset of cells within a particular GAL4 expression pattern (Bohm *et al.* 2010; Fore *et al.* 2011; Sivanantharajah and Zhang 2015). This technique uses the extensive collection of GAL4-UAS reagents already available and integrates two key additions: Flippase-mediated *GAL80* transgenes and a large collection of enhancer-trap Flippase lines (ET-FLPx2 lines). The ET-FLPx2 lines express Flippase (Flp) in subsets of cells within developing tissues. When brought together with Flp-sensitive target transgenes that contain either the *GAL80* ORF flanked by Flp recognition target (FRT) sites or a STOP cassette flanked by FRT sites, it is possible to enable the expression/repression of GAL4 within an enhancer-GAL4 domain in those cells that express Flp. The FINGR method thus enables greater resolution of the existing GAL4-UAS arsenal by “Flp-out” or “Flp-in” of GAL80 expression within a given GAL4 expression domain.

Here, we expand the utility of the FINGR method by characterizing a large collection of ET-FLPx2 lines for their Flp expression patterns in the third larval instar imaginal discs. We describe several lines with Flp expression in developing larval tissues, and demonstrate the potential of these tools for developmental studies by manipulating cell polarity during wing development.

## Materials and Methods

### *Drosophila* stocks

All *Drosophila* stocks were maintained at 25° on standard cornmeal-molasses medium and a 12-hr light:dark diurnal cycle. The collection of ET-FLPx2 insertion lines each contain a single copy of a transgene with two *flp* ORFs separated by an internal ribosome entry site sequence (IRES; hence, the transgene sequence is *flp*-IRES-*flp*) derived from the *Ultrabithorax* locus (Halfon *et al.* 2002). Details of ET-FLPx2 transgene construction can be found in Bohm *et al.* (2010). A *yw*, *actin^P^ > CD2 > GAL4*; *UAS-GFP* stock (Pignoni and Zipursky 1997) was used to report evidence of Flp recombination events in larval tissues. The *CD2* sequence interrupts transcription from the *actin* promoter (*actin^P^*) to prevent expression of *GAL4* in this transgene, and is flanked by FRT sites (denoted by “>”) that enable removal of the *CD2* cassette by Flp. A *w*; *tubulin^P^ > GAL80>* stock (hereafter, “TG”; Gordon and Scott 2009) and a *w*; *tubulin^P^ > STOP > GAL80* (hereafter, “TSG”; Bohm *et al.* 2010) stock were used to perform GAL80 Flp-out or Flp-in experiments, respectively.

### ET-FLPx2 expression pattern screen

Males from each ET-FLPx2 line were crossed *en masse* to *yw*, *actin^P^ > CD2 > GAL4*; *UAS-GFP* females. Their progeny were collected as wandering third instar larvae and sexed using morphological differences in the developing germline and genital imaginal disc. For each line we studied, we dissected all eye discs, wing discs, haltere discs, leg discs, and genital discs (13 discs total per individual) from a minimum of 10 males and 10 females. Immediately after dissection, imaginal discs were fixed in 4% paraformaldehyde in 1× phosphate-buffered saline using standard protocols. Discs were mounted in glycerol and imaged at 100× magnification (200× magnification for genital discs) using a Zeiss Imager.M2 microscope equipped with an ApoTome.2 and AxioCam MRm digital camera to provide high-resolution structural illumination. Each disc image was obtained using both bright field differential interference contrast and fluorescence using Zeiss filter set 38 Endow GFP shift free (excitation band pass = 470 nm, emission band pass = 525 nm). Digital images were overlaid and rendered using AxioVision software version 4.8.2. For those ET-FLPx2 lines that showed green fluorescent protein (GFP) expression in imaginal tissues, we crossed each line to *w*; *wg^Sp^/CyO*; *Sens^Ly-1^/TM6B,Tb* to identify the chromosome on which the *ET-FLPx2* transgene resides.

To characterize ET-FLPx2 expression patterns in the larval central nervous system (CNS), we dissected the brain and ventral nerve cord from *yw*, *actin^P^ > CD2 > GAL4*; *UAS-GFP*; *ET-FLPx2* third instar larvae; fixed them immediately by using 4% paraformaldehyde in 0.1 M Tris-HCl, 0.3M NaCl, and 0.5% Triton-X; then mounted and imaged these tissues as described previously. We quantified the number of GFP-expressing neurons by counting the number of cells in serial 1.5-μm thick sections through the entire larval brain. Cell bodies that showed overlap between adjacent optical sections were scored as a single cell when we calculated the total cell count.

### FINGR method in larval imaginal tissues

We selected three GAL4 lines that possess different spatial expression patterns in the third instar wing disc to demonstrate the FINGR method: *apterous-GAL4* (*apGAL4*), which is expressed broadly in the dorsal domain of the wing disc (Cohen *et al.* 1992); *nubbin-GAL4* (*nubGAL4*), which is expressed throughout the wing pouch (Cifuentes and García-Bellido 1997); and *vestigial-GAL4* (*vgGAL4*), which is expressed in a band of cells that extends through the medial region of the wing pouch (Williams *et al.* 1994). These GAL4 lines were used to construct *ET-FLPx2*; *TG*; *GAL4*, *UAS-GFP* and *ET-FLPx2*; *TSG*; *GAL4*, *UAS-GFP* genotypes to generate and visualize intersectional GAL80 Flp-out and Flp-in patterns in the wing disc, respectively. Imaginal discs were dissected, prepared, and imaged as described previously.

To demonstrate the potential of the FINGR method for enabling investigation of cell-specific contributions to adult morphologic structures, we knocked down expression of a key regulator of planar cell polarity, *prickle* (*pk*; Gubb and García-Bellido 1982) via RNA interference, to assay bristle polarity defects in the adult wing. We generated TG and TSG genotypes using *nubGAL4* (or *vgGAL4*), *ET-FLPx2*, and *UAS-pkIR*, which produces a RNA hairpin against *pk*. We dissected the left and right wings from 12 progeny from TG and TSG crosses each, and mounted the wings in glycerol. One wing from each individual we studied was chosen at random to quantify the wing compartments that showed bristle polarity defects.

### Data availability

The data associated with this article have been deposited in the Dryad Digital Repository: http://dx.doi.org/10.5061/dryad.6rh3p.

## Results and Discussion

### ET-FLP expression in imaginal tissues

To identify Flp recombination events in the imaginal discs, we crossed 521 ET-FLPx2 lines individually to *yw*, *actin^P^ > CD2 > GAL4*; *UAS-GFP*. In the progeny, cells that express Flp catalyze the removal of the *CD2* stop cassette and enable GFP expression. We found 28 of the 521 (5.4%) ET-FLPx2 lines that we screened show evidence of Flp recombination in third instar imaginal discs (Table 1). Most of these imaginal disc ET-FLPx2 lines also showed GFP expression in other larval tissues such as the CNS, intestinal tract, and trachea. For two lines, however, expression was specific to imaginal discs at the third instar stage (lines 705A and 845B). We also found two lines that show sex-specific expression in female imaginal tissues (244A and 934B). Nineteen of 28 (68%) of these imaginal disc ET-FLPx2 lines show GFP expression in all five of the discs we screened, and only six lines (21%) show expression in a single type of imaginal disc (Table 1).

**Table 1:** Flippase expression in ET-FLPx2 imaginal tissues

ET-FLP Line	Transgene Location (Chromosome)	Eye Disc	Wing Disc	Haltere Disc	Leg Disc	Genital Disc	Sex-Specific Expression
36A*^a^*	2	X	X	X	X	X	
187A*^a^*	3	X	X	X	X	X	
232B	3	X	X	X	X	X	
244A	2					X	X
262A	2	X	X	X	X		
361A	3	X	X	X	X	X	
382A	2	X	X	X	X	X	
615C	3		X	X	X		
656A	2				X		
688A	3	X	X	X	X	X	
700A	2	X	X	X	X	X	
705A	2		X				
843B	3		X				
845B	3		X				
866A	3	X	X	X	X	X	
868A	2				X	X	
874B	3	X	X	X	X	X	
896A	3	X	X	X	X	X	
907A	2		X		X	X	
934B	ND				X		X
937A	2	X	X	X	X	X	
955A	2	X	X	X	X	X	
961B	2	X	X	X	X	X	
1002A	ND	X	X	X	X	X	
1005B	2	X	X	X	X	X	
1023A*^a^*	2	X	X	X	X	X	
1030B	3	X	X	X	X	X	
1040A	3	X	X	X	X	X	
1107A	3	X	X	X	X	X	

ET-FLP, enhancer-trap Flippase; X, FLP expression; ND, not determined.

a Lines with reversed TG and TSG patterns.

**Figure 1:**
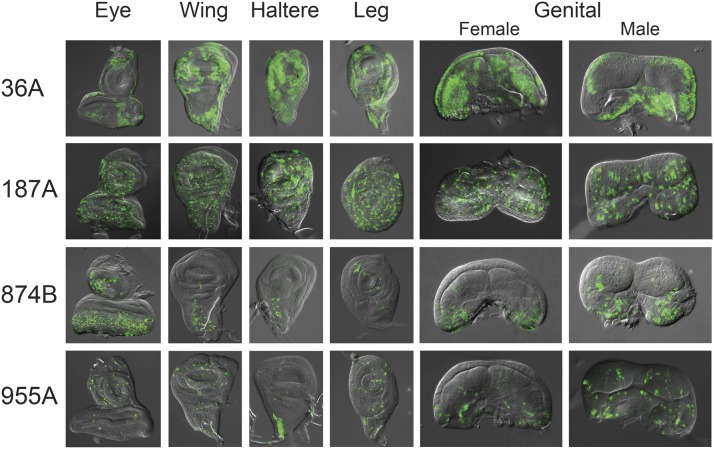
Expression patterns that result from Flippase (Flp) recombination events in developing imaginal discs. Evidence of Flp recombination events was visualized by crossing each ET-FLPx2 line to *actin^P^ > CD2 > GAL4*; *UAS-GFP*. Cells that express Flp catalyze the removal of the stop cassette *CD2* to allow expression of green fluorescent protein.

Figure 1 shows the variety of expression patterns we observed among the ET-FLPx2 lines. The expression patterns range from localized clusters of cells similar in appearance to those produced in heat-shock clonal analyses, to more restricted and “speckled” cell patterns within the tissues. Within a given line, the GFP expression pattern appears qualitatively similar among all five types of discs we examined. In particular, transgenes that produced GFP expression in localized clusters of cells showed a similar pattern across all five types of imaginal discs (*e.g.*, 36A, Figure 1), whereas transgenes that produced speckled GFP patterns did so across all discs (*e.g.*, 187A, Figure 1). Paired discs (*e.g.*, wing, haltere, leg) showed similar expression patterns within individuals, and we also observed similar expression patterns among all three pairs of leg discs. In general, GFP expression patterns appear consistent for each line, although there is expression variability within some ET-FLPx2 lines (Supporting Information, Figure S1). Representative expression patterns from imaginal disc ET-FLPx2 lines are available in the Dryad Digital Repository (doi: 10.5061/dryad.6rh3p).

Although GFP expression in the third instar imaginal discs marks those cells that lack the *CD2* stop cassette from the original *actin^P^ > CD2 > GAL4* transgene, it is important to note that the GFP patterns we used to characterize the ET-FLPx2 lines might not be an indicator of active Flp expression in these cells. Because Flp excises *CD2*, any daughter cells of earlier progenitors that experienced Flp recombination also will express GFP. This scenario is consistent with the observation of clusters of GFP-expressing cells in some of the ET-FLPx2 lines. In these cases, Flp expression may have occurred during the first or second larval instar stage and gave rise to clusters of daughter cells in the third instar discs that inherited the *actin^P^ > GAL4* allele. This type of event may also explain why we observe some variation in GFP expression patterns among individuals within lines. There are two additional possibilities that might also explain the variation in GFP expression patterns we observed in some ET-FLPx2 lines. One possibility is that variation in expression patterns among individuals could occur as a consequence of individual variation in Flp expression from the enhancer trap. Variation in Flp levels is known to affect recombination efficiency (*e.g.*, Schebelle *et al.* 2010), and some *ET-FLPx2* transgenes could reside in genomic regions that make them subject to position effect variegation for Flp expression. Another possible explanation for varying GFP expression patterns among individuals of a single ET-FLPx2 line is that these particular inserts might express Flp both pre- *and* post-mitotically at this developmental stage (Bohm *et al.* 2010). Bearing these possibilities in mind, we refer to cells that possess Flp recombination events as “Flp-expressing” throughout the remainder of the text to simplify the explanation of our experimental results.

### Intersectional GAL80 Flp-out and Flp-in approach

**Figure 2:**
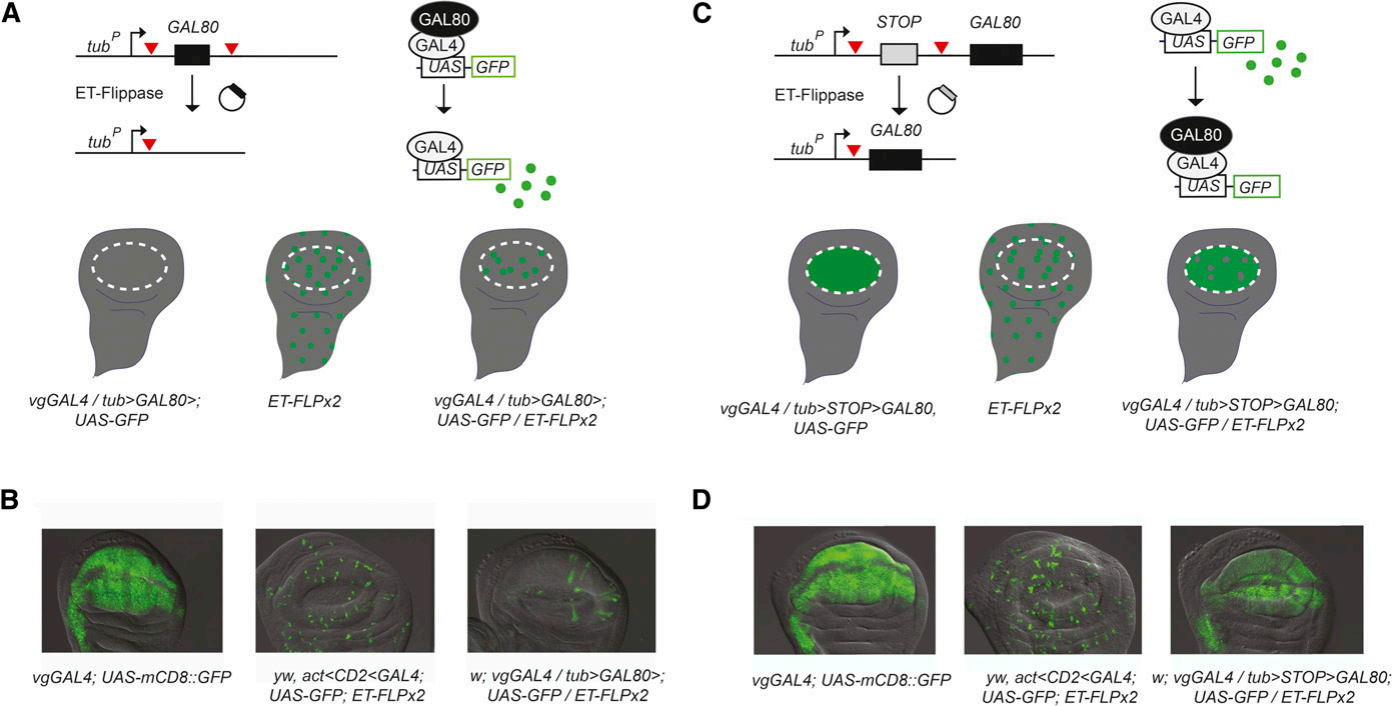
**F**lippase-induced **in**tersectional **G**AL80/GAL4 **r**epression (FINGR) method in the wing disc. (A) In *ET-FLPx2*; *TG*; *GAL4-UAS-GFP* genotypes, Flippase (Flp) catalyzes the permanent removal of *GAL80* for Flp-out expression of *UAS-GFP*. (B) Example of the *w*; *tubulin^P^ > GAL80>*(TG) method using ET-FLPx2 line 955A. The left panel shows vgGAL4 expression pattern, the middle panel shows ET-FLPx2 Flp-induced expression pattern, and the right panel shows the TG intersectional result. (C) In *ET-FLPx2*; *TSG*; *GAL4-UAS-GFP* genotypes, Flp catalyzes the permanent removal of the *STOP* cassette for Flp-in repression of *UAS-GFP*. (D) Example of *w*; *tubulin^P^ > STOP > GAL80* (TSG) method using ET-FLPx2 line 955A. The left panel shows vgGAL4 expression pattern, the middle panel shows ET-FLPx2 Flp-induced expression pattern, and the right panel shows the TSG intersectional result.

Figure 2 shows the expected GAL4 expression patterns in the wing imaginal disc produced via use of the FINGR method with vgGAL4 and ET-FLPx2 955A, which possesses a speckled Flp expression pattern within the vgGAL4 domain. In the TG cross (Figure 2, A and B), cells that express Flp experience the removal of the *GAL80* ORF from the *TG* transgene, which results in GAL80 repression of GAL4 activity in all vgGAL4 cells except those cells that express Flp, and only the Flp-expressing cells that reside within the vgGAL4 domain express GAL4. In the TSG cross (Figure 2, C and D), cells that express Flp experience the removal of the *STOP* cassette from the *TSG* transgene. This results in GAL80 repression of GAL4 activity only in Flp-expressing cells that reside within the vgGAL4 domain; all other cells in the vgGAL4 domain express GAL4. In general, for ET-FLPx2 lines that possess Flp expression patterns similar to 955A, TG crosses produce more restricted GAL4 expression within the enhancer-GAL4 domain, whereas TSG crosses produce broader GAL4 expression within the enhancer-GAL4 domain.

**Figure 3:**
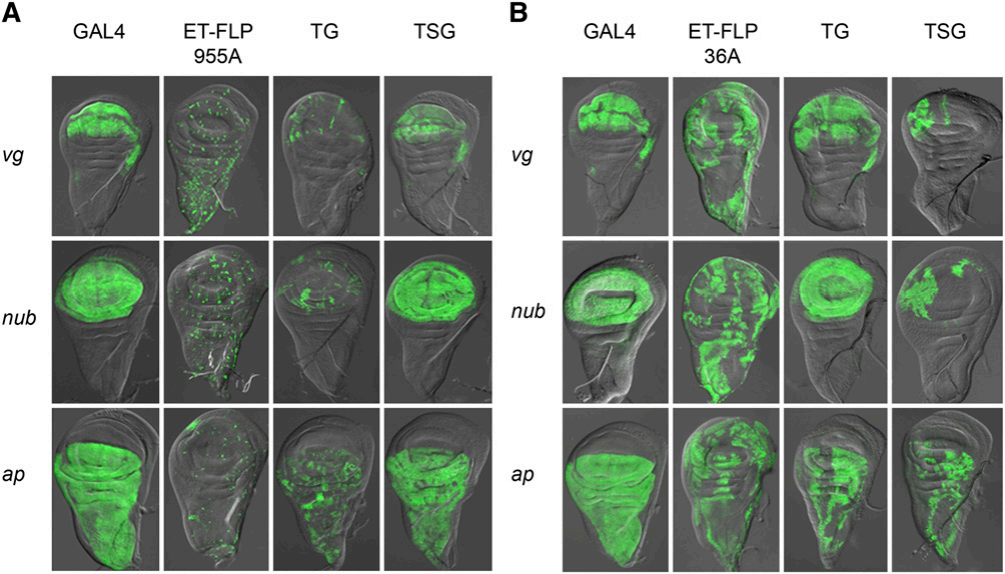
*w*;*tubulin^P^ > GAL80>*(TG) and *w*; *tubulin^P^ > STOP > GAL80* (TSG) expression patterns remain consistent using different GAL4 drivers. GAL4 expression patterns from TG and TSG crosses using three different GAL4 drivers in the wing imaginal disc. (A) ET-FLPx2 line 955A. (B) ET-FLPx2 line 36A.

We tested the ET-FLPx2 lines using three different GAL4 drivers to assess the broad utility of the collection of imaginal disc ET-FLPx2 lines for Flp-out and Flp-in experiments. We found that most ET-FLPx2 lines produce the expected GAL4 expression patterns within the GAL4 domain for each of the three GAL4 drivers we tested (Figure 3A). This was true for lines that produce speckled Flp expression patterns and also for those that produce clustered Flp expression patterns. Surprisingly, however, three ET-FLPx2 lines that we tested showed TG and TSG GAL4 expression patterns that appeared opposite of those expected (Figure 3B and Figure S2). Specifically, TG crosses with these lines result in patterns consistent with those expected from TSG crosses and vice versa. We obtained these same reversed results using all three GAL4 drivers.

Because the initial characterization of the ET-FLPx2 resource and the FINGR method were performed in CNS tissues (Bohm *et al.* 2010), we performed TG and TSG crosses using one of these “reversed” ET-FLPx2 lines and characterized GAL4 expression in the third instar larval brain to determine whether the Flp-out and Flp-in patterns were also reversed in neural tissues. We used the *nubGAL4* driver to perform these crosses; *nubbin* is expressed in a subset of dopaminergic and seratonergic neurons in the larval brain (Lundell and Hirsh 1998) in addition to larval wing disc expression. Our results using ET-FLPx2 187A for FINGR in the larval CNS produced GAL4 expression patterns that are consistent with those expected from TG and TSG crosses (Figure 2 and Figure S3), and thus show that these ET-FLPx2 lines behave as expected in neural tissues.

Although it is unclear exactly why these three ET-FLPx2 inserts behave opposite to what is expected in imaginal disc tissues, one possible explanation might have to do with the level of Flp expression in imaginal disc cells. The ET-FLPx2 transgene contains a *flp*-IRES-*flp* sequence, which was designed specifically to increase Flp expression in neurons (see Bohm *et al.* 2010). Should the ET-FLPx2 transgenes in these three lines reside in genomic regions that are highly transcribed in imaginal discs, this could give rise to particularly high Flp levels within imaginal disc cells. At very high titers of Flp, it seems possible that the reverse reaction might compete and enable reinsertion of the excised cassette. It is unknown how Flp titers affect recombinase function *in vivo*, and regardless of the ultimate functional explanation for these reversed patterns, we found that these three lines still prove to be valuable tools for manipulating gene expression in imaginal tissues (see *FINGR facilitates genetics manipulation of localized regions with GAL4 expression patterns*).

### FINGR facilitates genetic manipulation of localized regions within GAL4 expression patterns

The cells that reside within the wing pouch of the third instar wing imaginal disc ultimately give rise to the proximal and distal regions of the adult wing blade and parts of the wing hinge (Bryant 1975; Figure S4). The collection of existing GAL4 drivers that are expressed in the wing pouch often have broad expression domains (*e.g.*, Figure 3), which makes it difficult to target specific wing compartments to study the effects of gene misexpression, as most gene manipulation experiments using these broadly expressed GAL4 drivers severely abrogate wing development. To demonstrate the potential for the FINGR method to refine existing GAL4 expression patterns and make it possible to study localized sections of wing tissues, we used ET-FLPx2 36A to target clusters of cells in which to reduce expression of the PCP gene *pk* during wing development. *pk* is a major regulator of epithelial cell polarity (Shulman *et al.* 1998); in wing discs, *pk* is expressed at high levels within the wing pouch and expression persists into pupal development, where *pk* transcripts localize to the cells of all intervein compartments of the developing wing (Gubb *et al.* 1999).

**Figure 4:**
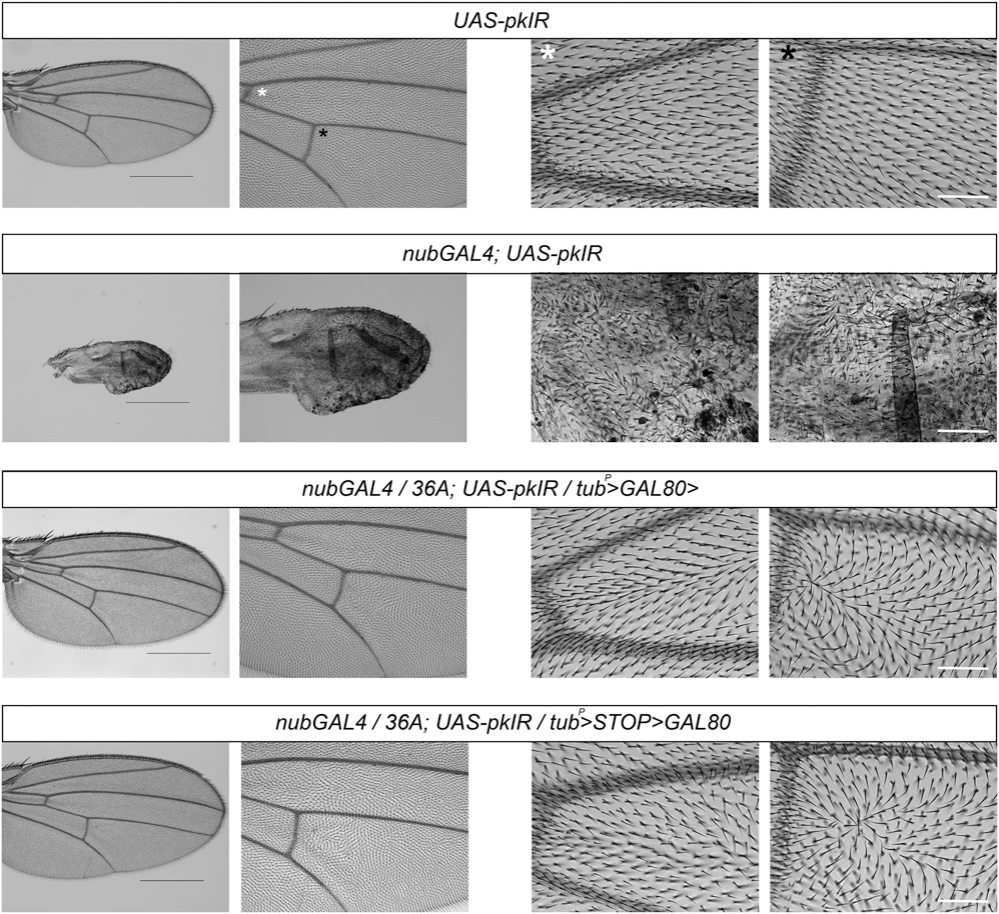
*w*;*tubulin^P^ > GAL80>*(TG) and *w*; *tubulin^P^ > STOP > GAL80* (TSG) crosses enable investigation of bristle polarity defects in the developing wing. From left to right: First panel shows an entire adult wing at 25× magnification. Scale bar is 500 µm. The second panel shows the same wing at 50× magnification. The final two panels show wing compartments at 100× magnification. Scale bar is 50 µm. White asterisks denote the region at the intersection of wing vein L3 and the anterior crossvein, and black asterisks denote the region at the intersection of wing vein L4 and the posterior crossvein.

We generated *nubGAL4*; *UAS-pkIR* individuals to reduce *pk* transcript levels in the wing pouch during larval development. (The *UAS-pkIR* transgene produces a RNA hairpin to induce RNA interference in the presence of GAL4.) Our results show that knockdown of *pk* severely abrogates wing development and results in adults with vestigial wings (Figure 4). These *nubGAL4*; *UAS-pkIR* individuals also exhibit severe polarity defects in wing cells and lack clearly identifiable wing venation patterns. We generated TG and TSG genotypes using ET-FLPx2 36A to restrict *pk* knockdown to more localized regions within the developing wing to rescue the severe developmental defects in *nubGAL4*; *UAS-pkIR* wings, and produce wings of relatively normal size and shape that possess clusters of wing compartment cells that exhibit polarity defects. Based on the 36A GFP expression patterns and the reversed GAL4 expression patterns identified in the TG and TSG crosses using 36A described previously, we predicted that knockdown of *pk* in TG and TSG patterns would produce wings with similar proportions of tissue that exhibit polarity defects, but that the regions of affected wing tissue would differ between the TG and TSG crosses. In particular, we expected TG *pk* knockdown to primarily affect proximal wing blade cells at the anterior (A) and posterior (P) regions of the adult wing, and TSG *pk* knockdown to primarily affect proximal wing blade cells that were localized more medially at the A/P boundary of the wing. Both TG and TSG crosses were expected to produce similar phenotypes in the distal portions of the adult wing.

We found that restricting *pk* knockdown to localized patches of cells in both TG and TSG crosses rescued wing development compared with the *nubGAL4*; *UAS-pkIR* genotype (Figure 4). When we compared the locations of the wing compartments with bristle polarity defects, we also observed the expected phenotypic differences between progeny of the TG and TSG crosses: TSG progeny possessed polarity defects in the compartments that are located medially near the A/P boundary of the proximal wing blade, whereas TG progeny do not (Figure 4). We obtained similar results when we performed TG and TSG *pk* knockdown using the *vgGAL4* driver, which produces less severe *vgGAL4*; *UAS-pkIR* developmental defects compared to *nubGAL4*; *UAS-pkIR* (Figure S5). Quantification of the individual wing compartments that exhibit polarity defects also show that compartments possessing bristle polarity defects are relatively equal in total number between the TG and TSG crosses, and that compartments in the distal wing are equally affected in both (Figure S6). However, in the proximal regions of the wing, the TG cross produced polarity defects in the A and P regions, whereas the TSG cross produced polarity defects in the medial region (Figure S6).

We identified and characterized several enhancer-trap Flp lines for use in larval imaginal discs, and the results of our experiments show that FINGR is a powerful method that enables refinement of the GAL4 expression patterns within the existing collection of GAL4-UAS tools in *Drosophila*. The ET-FLPx2 lines also offer several other advantages for expanding genetic manipulation studies in imaginal discs: 1) The ET-FLPx2 lines can be used with other Flp-mediated gene manipulation approaches, such as mosaic analysis with a repressible cell marker (*e.g.*, MARCM method; Lee and Luo 1999). 2) The patterns of Flp expression among the ET-FLPx2 lines can be used to generate “ET-FLPx2” GAL4 drivers when combined with a constitutively-expressed GAL4 driver and TG. 3) The ET-FLPx2 lines can facilitate clonal analysis without some of the complications of standard approaches (*e.g.*, heat shock treatment) and has been used successfully to generate clones in the *Drosophila* germline (Huang *et al.* 2014).

## 
